# 5,10,15,20-Tetra­kis(4-acetyl­oxyphen­yl)porphyrin including an unknown solvate

**DOI:** 10.1107/S1600536812047332

**Published:** 2012-11-28

**Authors:** Micael D. Miranda, Manuela Ramos Silva, Teresa M. R. Maria, Avula Balakrishna, Abilio J. F. N. Sobral

**Affiliations:** a CEMDRX, Physics Department, University of Coimbra, P-3004-516 Coimbra, Portugal; b Chemistry Department, University of Coimbra, P-3004 Coimbra, Portugal

## Abstract

Mol­ecules of the title compound, C_52_H_38_N_4_O_8_, are located on an inversion center so that the asymmetric cell contains one half of the mol­ecule. The macrocycle exhibits a ruffled conformation with a maximum deviation of 0.16 Å for the 24 macrocycle atoms: the dihedral angle between adjacent five-membered rings is 5.13 (19)°. The benzene rings are rotated by 70.25 (19)° with respect to their adjacent protonated five-membered rings, and by 65.56 (19)° with respect to the unprotonated rings. The porphyrin conformation is supported by bifurcated N—H⋯(N,N) hydrogen bonds. The structure contained poorly resolved solvent mol­ecules in voids of volume 217 Å^3^ per unit cell. The latter were treated using the SQUEEZE routine in *PLATON* [Spek (2009 ▶). *Acta Cryst*. D**65**, 148–155]. As the solvent could not be identified exactly, it was not included in the calculation of the overall formula weight, density and absorption coefficient.

## Related literature

For general background on porphyrin and porphyrin precursors synthesized in our group, see Paixão, Matos Beja *et al.* (2002 ▶); Paixão, Ramos Silva *et al.* (2002 ▶); Paixão *et al.* (2003 ▶); Ramos Silva *et al.* (2002*a* ▶,*b* ▶); Sobral *et al.* (2001*a* ▶,*b* ▶). For the applications of porphyrins, see: Zhang *et al.* (2010 ▶); Eichhorn (2000 ▶).
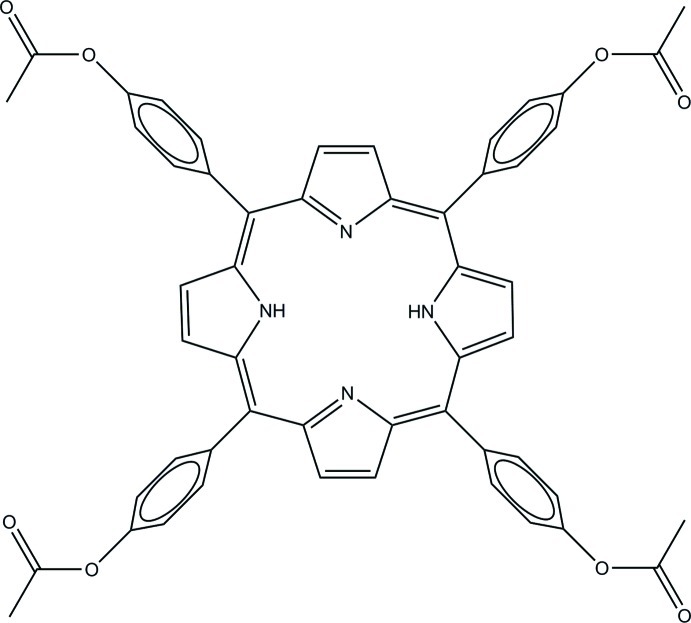


## Experimental

### 

#### Crystal data

C_52_H_38_N_4_O_8_
*M* *_r_* = 846.86
Triclinic, 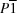

*a* = 6.6203 (2) Å
*b* = 14.1043 (3) Å
*c* = 14.4936 (3) Å
α = 113.862 (1)°
β = 97.771 (2)°
γ = 98.060 (2)°
*V* = 1197.58 (5) Å^3^
*Z* = 1
Mo *K*α radiation
μ = 0.08 mm^−1^
*T* = 293 K
0.30 × 0.12 × 0.04 mm

#### Data collection

Bruker APEX CCD area-detector diffractometer
Absorption correction: multi-scan (*SADABS*; Sheldrick, 2000 ▶) *T*_min_ = 0.790, *T*_max_ = 0.999
24062 measured reflections
4566 independent reflections
2956 reflections with *I* > 2σ(*I*)
*R*_int_ = 0.031

#### Refinement

*R*[*F* ^2^ > 2σ(*F* ^2^)] = 0.080
*wR*(*F* ^2^) = 0.248
*S* = 1.14
4566 reflections
291 parameters
H-atom parameters constrained
Δρ_max_ = 0.98 e Å^−3^
Δρ_min_ = −0.51 e Å^−3^

### 

Data collection: *SMART* (Bruker, 2003 ▶); cell refinement: *SAINT* (Bruker, 2003 ▶); data reduction: *SAINT*; program(s) used to solve structure: *SHELXS97* (Sheldrick, 2008 ▶); program(s) used to refine structure: *SHELXL97* (Sheldrick, 2008 ▶); molecular graphics: *ORTEPII* (Johnson, 1976 ▶); software used to prepare material for publication: *SHELXL97*.

## Supplementary Material

Crystal structure: contains datablock(s) global, I. DOI: 10.1107/S1600536812047332/hb6985sup1.cif

Structure factors: contains datablock(s) I. DOI: 10.1107/S1600536812047332/hb6985Isup2.hkl

Additional supplementary materials:  crystallographic information; 3D view; checkCIF report

## Figures and Tables

**Table 1:** Hydrogen-bond geometry (Å, °)

*D*—H⋯*A*	*D*—H	H⋯*A*	*D*⋯*A*	*D*—H⋯*A*
N1—H1⋯N2	0.86	2.35	2.885 (3)	121
N1—H1⋯N2^i^	0.86	2.42	2.944 (3)	120

Symmetry code: (i) 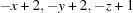.

**Fig. 1.**
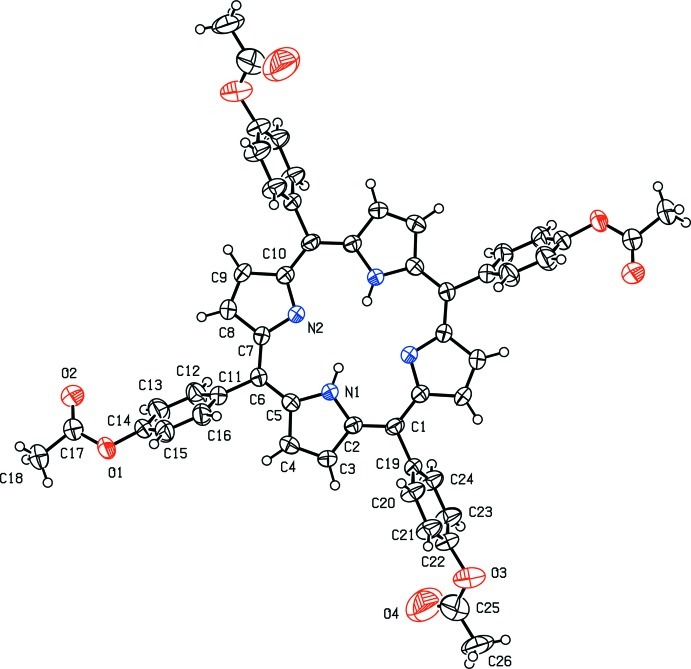
ORTEPII (Johnson, 1976) plot of the title compound. Displacement ellipsoids are drawn at the 50% level.

**Fig. 2.**
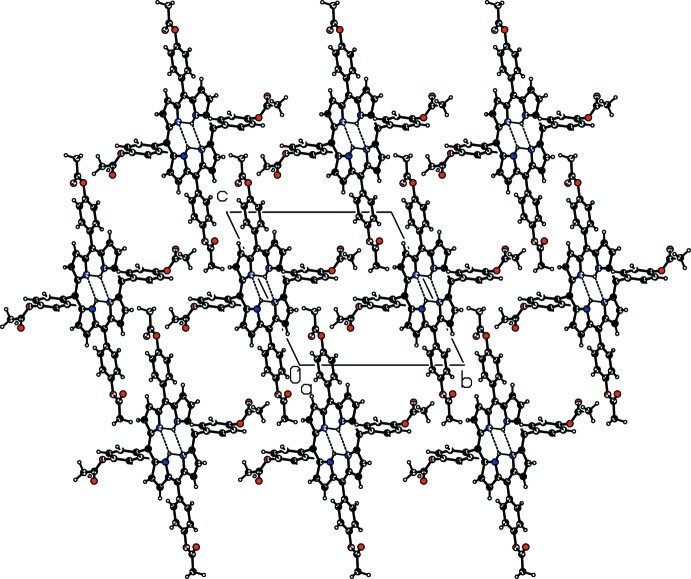
Packing of the molecules in the unit cell showing the H-bonds as dashed lines.

## supplementary crystallographic information

### Comment

This work is part of a project of synthesizing porphyrins and porphyrin percursors (Paixão, Matos Beja *et al.*, 2002; Paixão, Ramos Silva *et al.*, 2002; Paixão *et al.*, 2003; Ramos Silva *et al.*, 2002*a*,*b*; Sobral *et al.*, 2001*a*,*b*). Our aim is to use the tremendous potential for the manifold applications of porphyrins and obtain molecular magnets (Zhang *et al.*, 2010), liquid crystals (Eichhorn, 2000), multi-porous materials for CO_2_ sequestering and possibly some properties combined. In the title compound, Fig. 1, 5,10,15,20-Tetrakis[(4-methylcarbonyl)oxy]-phenylporphyrin, the porphyrin moiety shows a non planar ruffled conformation. The phenyl rings are rotated with respect to the porphyrin ring. The angle between the least-squares plane containing the porphyrin core and the least-squares plane of the phenyl ring C11—C16 is 65.29 (14)° [74.18 (13)° for C19—C24]. Intramolecular N—H···N hydrogen bonds are present. The molecules pile in columns along the *a* axis. There are solvent acessible voids in the crystal structure that accomodate solvent molecules in a very disordered way; these solvent molecules were not included in the calculation of the overall formula weight, density and absorption coefficient.

### Experimental

All reagents were used as purchased, except pyrrole that was distillated under reduced pressure. The tetrakys-(pheny-4-acetate)-21H,22*H*-porphine was synthesized by the method of Rothemund/Adler/Long [1–2]. The aldehyde 4-formylphenyl acetate (1 g, 6.1 mmol) was added to propionic acid (150 ml). The solution was placed to reflux and then pyrrole (0.5 ml, 7.207 mmol) was added drop wise during 10 minutes. The solution was left at 120 C for 4 h. The solvent was then evaporated and the crude, dissolved in dichloromethane, was washed with aqueous NaHCO3 and distilled water and dried with Na2SO4 anhydrous. The final porphyrin tetrakys-(pheny-4-acetate)-21H,22*H*-porphine was obtained after purification by column chromatography in silica/dichloromethane. Recrystallization in dichloromethane/hexane gives the purple crystals of tetrakys-(pheny-4-acetate)-21H,22*H*-porphine (yield of 5% relatively to pyrrole). HPLC/MS showed a single signal corresponding to the expected molecular ion m/*z* 847.

### Refinement

H atoms bound to C atoms were placed at calculated positions and were treated as riding on the parent atoms with C—H = 0.93 Å (aromatic) and with *U*_iso_(H) = 1.2 *U*_eq_(C). In a final difference Fourier map highly disordered electron density occupying one cavity of *ca* 215 Å3 each was observed. This residual electron density was difficult to model and therefore, the SQUEEZE routine in *PLATON* (Spek, 2009) was used to eliminate this contribution of the electron density in the solvent region from the intensity data. The solvent-free model was employed for the final refinement. One of the methylcarbonyloxy terminal groups shows signs of disorder, possibly occupying two or more sites but such disorder could not be resolved in the present experiment. The solvent molecules were not included in the calculation of the overall formula weight, density and absorption coefficient.

### Crystal data

**Table:**

C_52_H_38_N_4_O_8_	*Z* = 1
*M**_r_* = 846.86	*F*(000) = 442
Triclinic, *P*1	*D*_x_ = 1.174 Mg m^−^^3^
*a* = 6.6203 (2) Å	Mo *K*α radiation, λ = 0.71073 Å
*b* = 14.1043 (3) Å	Cell parameters from 4592 reflections
*c* = 14.4936 (3) Å	θ = 2.9–21.4°
α = 113.862 (1)°	µ = 0.08 mm^−^^1^
β = 97.771 (2)°	*T* = 293 K
γ = 98.060 (2)°	Plate, violet
*V* = 1197.58 (5) Å^3^	0.30 × 0.12 × 0.04 mm

### Data collection

**Table:**

Bruker APEX CCD area-detector diffractometer	4566 independent reflections
Radiation source: fine-focus sealed tube	2956 reflections with *I* > 2σ(*I*)
Graphite monochromator	*R*_int_ = 0.031
φ and ω scans	θ_max_ = 25.8°, θ_min_ = 1.6°
Absorption correction: multi-scan (*SADABS*; Sheldrick, 2000)	*h* = −8→8
*T*_min_ = 0.790, *T*_max_ = 0.999	*k* = −17→17
24062 measured reflections	*l* = −17→17

### Refinement

**Table:**

Refinement on *F*^2^	Primary atom site location: structure-invariant direct methods
Least-squares matrix: full	Secondary atom site location: difference Fourier map
*R*[*F*^2^ > 2σ(*F*^2^)] = 0.080	Hydrogen site location: inferred from neighbouring sites
*wR*(*F*^2^) = 0.248	H-atom parameters constrained
*S* = 1.14	*w* = 1/[σ^2^(*F*_o_^2^) + (0.1221*P*)^2^ + 0.5589*P*] where *P* = (*F*_o_^2^ + 2*F*_c_^2^)/3
4566 reflections	(Δ/σ)_max_ = 0.040
291 parameters	Δρ_max_ = 0.98 e Å^−^^3^
0 restraints	Δρ_min_ = −0.51 e Å^−^^3^

### Special details

**Table:**

Geometry. All e.s.d.'s (except the e.s.d. in the dihedral angle between two l.s. planes) are estimated using the full covariance matrix. The cell e.s.d.'s are taken into account individually in the estimation of e.s.d.'s in distances, angles and torsion angles; correlations between e.s.d.'s in cell parameters are only used when they are defined by crystal symmetry. An approximate (isotropic) treatment of cell e.s.d.'s is used for estimating e.s.d.'s involving l.s. planes.
Refinement. Refinement of *F*^2^ against ALL reflections. The weighted *R*-factor *wR* and goodness of fit *S* are based on *F*^2^, conventional *R*-factors *R* are based on *F*, with *F* set to zero for negative *F*^2^. The threshold expression of *F*^2^ > σ(*F*^2^) is used only for calculating *R*-factors(gt) *etc*. and is not relevant to the choice of reflections for refinement. *R*-factors based on *F*^2^ are statistically about twice as large as those based on *F*, and *R*- factors based on ALL data will be even larger.

### Fractional atomic coordinates and isotropic or equivalent isotropic displacement parameters (Å^2^)

**Table:**

	*x*	*y*	*z*	*U*_iso_*/*U*_eq_	
N1	0.7700 (3)	0.90482 (18)	0.36681 (17)	0.0397 (6)	
H1	0.8553	0.9529	0.4212	0.048*	
N2	1.1829 (3)	1.03171 (17)	0.41002 (17)	0.0400 (6)	
O1	0.8965 (4)	0.7835 (2)	−0.19208 (16)	0.0709 (7)	
O2	1.2299 (4)	0.8442 (2)	−0.1891 (2)	0.0810 (8)	
O3	−0.1992 (4)	0.5305 (2)	0.3829 (3)	0.0907 (9)	
O4	−0.1461 (12)	0.4164 (5)	0.2427 (5)	0.270 (4)	
C1	0.5249 (4)	0.8381 (2)	0.4526 (2)	0.0404 (7)	
C2	0.5916 (4)	0.8419 (2)	0.3666 (2)	0.0399 (7)	
C3	0.4952 (4)	0.7749 (2)	0.2613 (2)	0.0477 (8)	
H3	0.3713	0.7240	0.2377	0.057*	
C4	0.6157 (5)	0.7981 (3)	0.2015 (2)	0.0511 (8)	
H4	0.5881	0.7664	0.1297	0.061*	
C5	0.7918 (4)	0.8795 (2)	0.2676 (2)	0.0418 (7)	
C6	0.9617 (4)	0.9196 (2)	0.2367 (2)	0.0414 (7)	
C7	1.1447 (4)	0.9906 (2)	0.3043 (2)	0.0419 (7)	
C8	1.3182 (5)	1.0335 (2)	0.2720 (2)	0.0513 (8)	
H8	1.3306	1.0176	0.2044	0.062*	
C9	1.4573 (5)	1.1004 (3)	0.3576 (2)	0.0511 (8)	
H9	1.5846	1.1402	0.3606	0.061*	
C10	1.3752 (4)	1.1001 (2)	0.4445 (2)	0.0410 (7)	
C11	0.9478 (5)	0.8834 (2)	0.1239 (2)	0.0453 (7)	
C12	1.0830 (5)	0.8245 (3)	0.0745 (2)	0.0601 (9)	
H12	1.1829	0.8067	0.1125	0.072*	
C13	1.0738 (6)	0.7915 (3)	−0.0297 (3)	0.0672 (10)	
H13	1.1658	0.7517	−0.0620	0.081*	
C14	0.9254 (5)	0.8187 (3)	−0.0849 (2)	0.0558 (9)	
C15	0.7864 (5)	0.8749 (3)	−0.0391 (2)	0.0579 (9)	
H15	0.6848	0.8913	−0.0777	0.070*	
C16	0.7987 (5)	0.9072 (3)	0.0653 (2)	0.0529 (8)	
H16	0.7046	0.9458	0.0969	0.063*	
C17	1.0572 (6)	0.7993 (3)	−0.2359 (3)	0.0599 (9)	
C18	0.9824 (6)	0.7539 (3)	−0.3500 (3)	0.0720 (11)	
H18A	0.9996	0.8098	−0.3718	0.108*	
H18B	0.8375	0.7197	−0.3682	0.108*	
H18C	1.0620	0.7030	−0.3834	0.108*	
C19	0.3332 (4)	0.7556 (2)	0.4305 (2)	0.0411 (7)	
C20	0.1366 (5)	0.7666 (3)	0.3969 (3)	0.0550 (8)	
H20	0.1215	0.8276	0.3888	0.066*	
C21	−0.0373 (5)	0.6893 (3)	0.3751 (3)	0.0620 (9)	
H21	−0.1685	0.6967	0.3505	0.074*	
C22	−0.0155 (5)	0.6014 (3)	0.3900 (3)	0.0575 (9)	
C23	0.1759 (5)	0.5883 (3)	0.4238 (3)	0.0658 (10)	
H23	0.1896	0.5280	0.4334	0.079*	
C24	0.3492 (5)	0.6658 (3)	0.4436 (3)	0.0575 (9)	
H24	0.4804	0.6570	0.4664	0.069*	
C25	−0.2590 (11)	0.4459 (5)	0.3054 (5)	0.119 (2)	
C26	−0.4600 (7)	0.3784 (4)	0.2982 (4)	0.1021 (16)	
H26A	−0.4679	0.3064	0.2502	0.153*	
H26B	−0.5742	0.4039	0.2748	0.153*	
H26C	−0.4677	0.3812	0.3649	0.153*	

### Atomic displacement parameters (Å^2^)

**Table:**

	*U*^11^	*U*^22^	*U*^33^	*U*^12^	*U*^13^	*U*^23^
N1	0.0341 (12)	0.0479 (12)	0.0322 (12)	−0.0020 (10)	0.0051 (9)	0.0165 (10)
N2	0.0370 (13)	0.0456 (12)	0.0342 (12)	−0.0015 (10)	0.0070 (10)	0.0176 (10)
O1	0.0544 (14)	0.1086 (19)	0.0343 (11)	−0.0056 (13)	0.0089 (10)	0.0232 (12)
O2	0.0619 (16)	0.0996 (19)	0.0612 (15)	−0.0139 (15)	0.0127 (13)	0.0248 (14)
O3	0.0685 (18)	0.0697 (16)	0.109 (2)	−0.0121 (14)	0.0007 (16)	0.0276 (16)
O4	0.299 (7)	0.196 (5)	0.175 (5)	−0.122 (5)	0.121 (5)	−0.023 (4)
C1	0.0297 (14)	0.0450 (14)	0.0472 (16)	0.0001 (12)	0.0055 (12)	0.0239 (12)
C2	0.0334 (14)	0.0447 (14)	0.0406 (15)	0.0011 (12)	0.0046 (11)	0.0207 (12)
C3	0.0365 (15)	0.0565 (17)	0.0412 (16)	−0.0064 (13)	0.0007 (12)	0.0199 (14)
C4	0.0438 (17)	0.0618 (18)	0.0358 (15)	−0.0013 (14)	−0.0014 (13)	0.0166 (14)
C5	0.0394 (15)	0.0483 (15)	0.0353 (14)	0.0039 (13)	0.0025 (12)	0.0191 (12)
C6	0.0424 (15)	0.0488 (15)	0.0327 (14)	0.0037 (13)	0.0065 (12)	0.0196 (12)
C7	0.0421 (16)	0.0486 (15)	0.0362 (14)	0.0010 (13)	0.0090 (12)	0.0221 (12)
C8	0.0513 (18)	0.0622 (18)	0.0382 (15)	−0.0011 (15)	0.0123 (13)	0.0229 (14)
C9	0.0425 (16)	0.0617 (18)	0.0452 (16)	−0.0079 (14)	0.0144 (13)	0.0236 (14)
C10	0.0362 (15)	0.0457 (14)	0.0408 (15)	0.0016 (12)	0.0069 (12)	0.0210 (12)
C11	0.0430 (16)	0.0534 (16)	0.0367 (15)	0.0012 (13)	0.0084 (12)	0.0198 (13)
C12	0.0515 (19)	0.085 (2)	0.0425 (17)	0.0186 (17)	0.0065 (14)	0.0258 (16)
C13	0.060 (2)	0.089 (3)	0.0459 (19)	0.0179 (19)	0.0127 (16)	0.0211 (18)
C14	0.0491 (18)	0.075 (2)	0.0317 (15)	−0.0069 (16)	0.0033 (13)	0.0195 (15)
C15	0.0530 (19)	0.079 (2)	0.0444 (17)	0.0081 (17)	0.0056 (14)	0.0324 (16)
C16	0.0493 (18)	0.0665 (19)	0.0443 (17)	0.0124 (15)	0.0132 (14)	0.0243 (15)
C17	0.060 (2)	0.067 (2)	0.0448 (18)	0.0034 (17)	0.0142 (16)	0.0192 (16)
C18	0.079 (3)	0.086 (3)	0.0442 (19)	0.010 (2)	0.0208 (18)	0.0222 (18)
C19	0.0344 (15)	0.0481 (15)	0.0400 (15)	−0.0001 (12)	0.0073 (11)	0.0214 (12)
C20	0.0360 (16)	0.0550 (17)	0.078 (2)	0.0039 (14)	0.0074 (15)	0.0366 (16)
C21	0.0350 (17)	0.067 (2)	0.085 (2)	0.0023 (15)	0.0050 (16)	0.0390 (18)
C22	0.0392 (17)	0.0559 (18)	0.068 (2)	−0.0092 (15)	0.0073 (15)	0.0252 (16)
C23	0.053 (2)	0.0539 (18)	0.092 (3)	−0.0018 (16)	0.0063 (18)	0.0406 (18)
C24	0.0377 (17)	0.0606 (19)	0.078 (2)	0.0025 (14)	0.0046 (15)	0.0387 (17)
C25	0.136 (5)	0.106 (4)	0.081 (3)	−0.023 (4)	0.005 (3)	0.027 (3)
C26	0.071 (3)	0.084 (3)	0.126 (4)	−0.032 (2)	0.001 (3)	0.042 (3)

### Geometric parameters (Å, º)

**Table:**

N1—C5	1.368 (4)	C11—C16	1.380 (4)
N1—C2	1.374 (3)	C11—C12	1.380 (5)
N1—H1	0.8600	C12—C13	1.378 (5)
N2—C7	1.372 (3)	C12—H12	0.9300
N2—C10	1.376 (3)	C13—C14	1.376 (5)
O1—C17	1.348 (4)	C13—H13	0.9300
O1—C14	1.402 (4)	C14—C15	1.364 (5)
O2—C17	1.187 (4)	C15—C16	1.379 (4)
O3—C25	1.226 (6)	C15—H15	0.9300
O3—C22	1.425 (4)	C16—H16	0.9300
O4—C25	1.235 (8)	C17—C18	1.490 (5)
C1—C2	1.395 (4)	C18—H18A	0.9600
C1—C10^i^	1.394 (4)	C18—H18B	0.9600
C1—C19	1.497 (4)	C18—H18C	0.9600
C2—C3	1.425 (4)	C19—C24	1.370 (4)
C3—C4	1.356 (4)	C19—C20	1.379 (4)
C3—H3	0.9300	C20—C21	1.373 (4)
C4—C5	1.424 (4)	C20—H20	0.9300
C4—H4	0.9300	C21—C22	1.365 (5)
C5—C6	1.396 (4)	C21—H21	0.9300
C6—C7	1.406 (4)	C22—C23	1.360 (5)
C6—C11	1.488 (4)	C23—C24	1.378 (4)
C7—C8	1.445 (4)	C23—H23	0.9300
C8—C9	1.333 (4)	C24—H24	0.9300
C8—H8	0.9300	C25—C26	1.489 (7)
C9—C10	1.439 (4)	C26—H26A	0.9600
C9—H9	0.9300	C26—H26B	0.9600
C10—C1^i^	1.394 (4)	C26—H26C	0.9600
			
C5—N1—C2	109.8 (2)	C15—C14—C13	121.4 (3)
C5—N1—H1	125.1	C15—C14—O1	115.9 (3)
C2—N1—H1	125.1	C13—C14—O1	122.5 (3)
C7—N2—C10	105.8 (2)	C14—C15—C16	119.0 (3)
C17—O1—C14	121.0 (3)	C14—C15—H15	120.5
C25—O3—C22	118.6 (5)	C16—C15—H15	120.5
C2—C1—C10^i^	126.2 (3)	C11—C16—C15	121.4 (3)
C2—C1—C19	116.0 (2)	C11—C16—H16	119.3
C10^i^—C1—C19	117.6 (3)	C15—C16—H16	119.3
N1—C2—C1	126.8 (2)	O2—C17—O1	124.2 (3)
N1—C2—C3	106.9 (2)	O2—C17—C18	126.3 (3)
C1—C2—C3	126.2 (3)	O1—C17—C18	109.5 (3)
C4—C3—C2	108.1 (2)	C17—C18—H18A	109.5
C4—C3—H3	126.0	C17—C18—H18B	109.5
C2—C3—H3	126.0	H18A—C18—H18B	109.5
C3—C4—C5	108.2 (3)	C17—C18—H18C	109.5
C3—C4—H4	125.9	H18A—C18—H18C	109.5
C5—C4—H4	125.9	H18B—C18—H18C	109.5
N1—C5—C6	126.7 (2)	C24—C19—C20	117.8 (3)
N1—C5—C4	107.0 (3)	C24—C19—C1	120.2 (3)
C6—C5—C4	126.2 (3)	C20—C19—C1	121.9 (3)
C5—C6—C7	124.7 (3)	C21—C20—C19	121.2 (3)
C5—C6—C11	117.3 (2)	C21—C20—H20	119.4
C7—C6—C11	118.0 (3)	C19—C20—H20	119.4
N2—C7—C6	125.5 (3)	C22—C21—C20	119.3 (3)
N2—C7—C8	110.0 (2)	C22—C21—H21	120.4
C6—C7—C8	124.5 (3)	C20—C21—H21	120.4
C9—C8—C7	106.8 (3)	C21—C22—C23	121.0 (3)
C9—C8—H8	126.6	C21—C22—O3	118.3 (3)
C7—C8—H8	126.6	C23—C22—O3	120.1 (3)
C8—C9—C10	107.8 (3)	C22—C23—C24	119.0 (3)
C8—C9—H9	126.1	C22—C23—H23	120.5
C10—C9—H9	126.1	C24—C23—H23	120.5
N2—C10—C1^i^	125.9 (3)	C23—C24—C19	121.6 (3)
N2—C10—C9	109.6 (2)	C23—C24—H24	119.2
C1^i^—C10—C9	124.6 (3)	C19—C24—H24	119.2
C16—C11—C12	117.9 (3)	O3—C25—O4	120.1 (6)
C16—C11—C6	121.1 (3)	O3—C25—C26	116.5 (6)
C12—C11—C6	121.0 (3)	O4—C25—C26	123.1 (5)
C13—C12—C11	121.7 (3)	C25—C26—H26A	109.5
C13—C12—H12	119.1	C25—C26—H26B	109.5
C11—C12—H12	119.1	H26A—C26—H26B	109.5
C14—C13—C12	118.5 (3)	C25—C26—H26C	109.5
C14—C13—H13	120.7	H26A—C26—H26C	109.5
C12—C13—H13	120.7	H26B—C26—H26C	109.5
			
C5—N1—C2—C1	174.5 (3)	C7—C6—C11—C12	63.6 (4)
C5—N1—C2—C3	−1.0 (3)	C16—C11—C12—C13	1.1 (5)
C10^i^—C1—C2—N1	1.9 (5)	C6—C11—C12—C13	−179.2 (3)
C19—C1—C2—N1	−175.0 (3)	C11—C12—C13—C14	0.1 (6)
C10^i^—C1—C2—C3	176.6 (3)	C12—C13—C14—C15	−1.5 (5)
C19—C1—C2—C3	−0.3 (4)	C12—C13—C14—O1	−176.2 (3)
N1—C2—C3—C4	0.2 (3)	C17—O1—C14—C15	131.0 (4)
C1—C2—C3—C4	−175.3 (3)	C17—O1—C14—C13	−54.0 (5)
C2—C3—C4—C5	0.6 (4)	C13—C14—C15—C16	1.5 (5)
C2—N1—C5—C6	−174.2 (3)	O1—C14—C15—C16	176.6 (3)
C2—N1—C5—C4	1.4 (3)	C12—C11—C16—C15	−1.1 (5)
C3—C4—C5—N1	−1.2 (4)	C6—C11—C16—C15	179.3 (3)
C3—C4—C5—C6	174.4 (3)	C14—C15—C16—C11	−0.2 (5)
N1—C5—C6—C7	2.8 (5)	C14—O1—C17—O2	−1.2 (6)
C4—C5—C6—C7	−171.9 (3)	C14—O1—C17—C18	179.9 (3)
N1—C5—C6—C11	−177.8 (3)	C2—C1—C19—C24	105.4 (3)
C4—C5—C6—C11	7.5 (5)	C10^i^—C1—C19—C24	−71.8 (4)
C10—N2—C7—C6	−177.7 (3)	C2—C1—C19—C20	−74.7 (4)
C10—N2—C7—C8	0.6 (3)	C10^i^—C1—C19—C20	108.2 (3)
C5—C6—C7—N2	−0.6 (5)	C24—C19—C20—C21	−1.3 (5)
C11—C6—C7—N2	179.9 (3)	C1—C19—C20—C21	178.7 (3)
C5—C6—C7—C8	−178.7 (3)	C19—C20—C21—C22	1.9 (6)
C11—C6—C7—C8	1.9 (5)	C20—C21—C22—C23	−1.4 (6)
N2—C7—C8—C9	−0.7 (4)	C20—C21—C22—O3	170.2 (3)
C6—C7—C8—C9	177.6 (3)	C25—O3—C22—C21	104.7 (5)
C7—C8—C9—C10	0.5 (4)	C25—O3—C22—C23	−83.6 (6)
C7—N2—C10—C1^i^	178.3 (3)	C21—C22—C23—C24	0.3 (6)
C7—N2—C10—C9	−0.3 (3)	O3—C22—C23—C24	−171.1 (3)
C8—C9—C10—N2	−0.1 (4)	C22—C23—C24—C19	0.3 (6)
C8—C9—C10—C1^i^	−178.7 (3)	C20—C19—C24—C23	0.2 (5)
C5—C6—C11—C16	63.8 (4)	C1—C19—C24—C23	−179.9 (3)
C7—C6—C11—C16	−116.7 (3)	C22—O3—C25—O4	10.6 (10)
C5—C6—C11—C12	−115.8 (3)	C22—O3—C25—C26	−175.6 (4)

Symmetry code: (i) −*x*+2, −*y*+2, −*z*+1.

### Hydrogen-bond geometry (Å, º)

**Table:**

*D*—H···*A*	*D*—H	H···*A*	*D*···*A*	*D*—H···*A*
N1—H1···N2	0.86	2.35	2.885 (3)	121
N1—H1···N2^i^	0.86	2.42	2.944 (3)	120

Symmetry code: (i) −*x*+2, −*y*+2, −*z*+1.
